# A SIFT Study of Reactions of Positive and Negative Ions With Polyfluoroalkyl (PFAS) Molecules in Dry and Humid Nitrogen at 393 K

**DOI:** 10.1002/rcm.9975

**Published:** 2024-12-26

**Authors:** Stefan James Swift, Maroua Omezzine Gnioua, Kseniya Dryahina, Patrik Španěl

**Affiliations:** ^1^ J Heyrovský Institute of Physical Chemistry of the CAS Prague 8 Czechia; ^2^ Faculty of Mathematics and Physics Charles University Prague 8 Czechia

**Keywords:** ion molecule reactions, perfluoroalkyl and polyfluoroalkyl substances (PFAS), selected ion flow tube

## Abstract

**Rationale:**

Data are required for SIFT‐MS analysis of perfluoroalkyl and polyfluoroalkyl substances (PFAS), which are persistent in the environment and cause adverse health effects. Specifically, the rate coefficients and product ion branching ratios of the reactions of H_3_O^+^, NO^+^, O_2_^+^•, O^−^•, OH^−^, O_2_^−^•, NO_2_^−^ and NO_3_^−^ with PFAS vapours are needed.

**Methods:**

The dual polarity SIFT‐MS instrument (Voice200) was used to generate these eight reagent ions and inject them into the flow tube with N_2_ carrier gas at a temperature of 393 K. Vapours of pentafluoropropionic acid, heptafluorobutyric acid, nonafluoro‐1‐hexanol, perfluoro‐2‐methyl‐2‐pentene, perfluorohexanoic acid, perfluoro(2‐methyl‐3‐oxahexanoic) acid, tridecafluoro‐1‐octanol and nonafluorobutane‐1‐sulfonic acid were introduced in dry and humid air. Full‐scan mass spectra were collected for all reagents at variable PFAS concentrations and analysed numerically.

**Results:**

Rate coefficients were determined for 64 reactions, for which 55 positive and 71 negative product ions were identified. The branching ratios for the primary reaction channels were extracted from the data, and the secondary chemistry with H_2_O molecules was qualitatively assessed. The thermochemical data were calculated for the H_3_O^+^ reactions using density functional theory (DFT).

**Conclusions:**

An important observation is that secondary reactions with water molecules remove the positive product ions, making them unsuitable for practical SIFT‐MS analysis of PFAS vapours. In contrast, most negative reaction product ions are not significantly affected by humidity and are thus preferred for the SIFT‐MS analyses of PFAS substances in various gaseous matrices.

## 1 Introduction

Selected ion flow tube mass spectrometry (SIFT‐MS) is a highly sensitive technique for the rapid analyses of trace gases and vapours present in air and other gaseous matrices [1, 2, 3]. This method is based on the chemical ionisation of a sample molecule by selectively filtered reagent ions injected into a flow tube, through which a fast flow of carrier gas passes. Helium was the most popular used carrier gas previously, but now it is routinely replaced by more readily available nitrogen [2, 4, 5]. The reagent ions can be rapidly switched between three cations (H_3_O^+^, NO^+^ and O_2_^+^•) and five anions (OH^−^, O_2_^−^•, O^−^•, NO_2_^−^ and NO_3_^−^) [6], and they react selectively with analyte molecules in the continuously flowing sample stream, during a well‐defined reaction time (typically 5 ms). The downstream quadrupole mass analyser with an electron multiplier detector measures the reagent and product ion signals according to their mass‐to‐charge ratios (*m/z*). The concentration of analyte molecules is subsequently calculated using the known ion‐molecule reaction rate coefficients and product ion branching ratios [2].

Polyfluorinated alkyl substances (PFAS) are a wide class of compounds [7] that are used in water‐repellent materials because of their hydrophobic nature [8], as well as in the production of household items [8]. Also known as ‘forever chemicals’ [9] when they are disposed of in the environment, they are persistent pollutants and an increasing public health issue [7] because of their toxicity [8, 10] and propensity to accumulate in the environment and food chain [9, 11]. Therefore, it is now important to develop reliable methods to analyse their presence in consumer products and other samples. PFAS are often analysed using chromatographic analyses (in some cases coupled to mass spectrometry), with sample run times on the order of minutes [12]. The ability to measure PFAS from the headspace of samples via online mass spectrometry would improve this sample run time to seconds. Recently, chemical ionisation mass spectrometry was developed to analyse PFAS in air with iodide [13, 14, 15] or acetate [16] reagent anions. However, SIFT‐MS has not yet been used extensively for PFAS analyses. As a first step in developing SIFT‐MS methods for PFAS analyses, it is important to understand the general features of their reactivity with the SIFT‐MS reagent ions.

Several previous studies have been carried out on reactions of negative ions with fluorinated hydrocarbons. Morris [17] studied the reactions of O_2_^−^• and O^−^• with C_2_F_4_, finding that F^−^ is the main product ion, whereas both of these reagent ions are unreactive with CF_4_. Later, a SIFT study was carried out in conjunction with ion mobility spectrometry (IMS) to detect toxic perfluoroisobutene (PFIB). This study revealed that the main reaction of O_2_^−^• with C_4_F_8_ is associative detachment, forming electrons that are not observed by SIFT, and that F^−^, COF^−^, CF_3_^−^, C_2_F_3_O^−^ and C_3_F_6_O^−^ are minor product ions (< 10%) [18]. Another SIFT study of OH^−^ with CH_2_F_2_ revealed that CHF_2_^−^ (86%), F^−^ (11%) and HF_2_^−^ (3%) are the main binary product ions and that a three‐body association reaction also occurs [19]. SIFT data are further available for the reactions of O_2_^−^•, O^−^• and OH^−^ with fluorinated ethenes, showing that C_2_F_4_ reacts with O_2_^−^˙ by associative detachment in parallel with F^−^ formation as the main ionic product, together with F_2_^−^, FCO^−^ and C_2_F_4_O^−^ as minor product ions. OH^−^ alternatively forms CF_3_^−^ as the main product ion with F^−^, HF_2_^−^, FCO^−^ and C_2_F_3_O^−^ as minor products [20].

**FIGURE 1:**
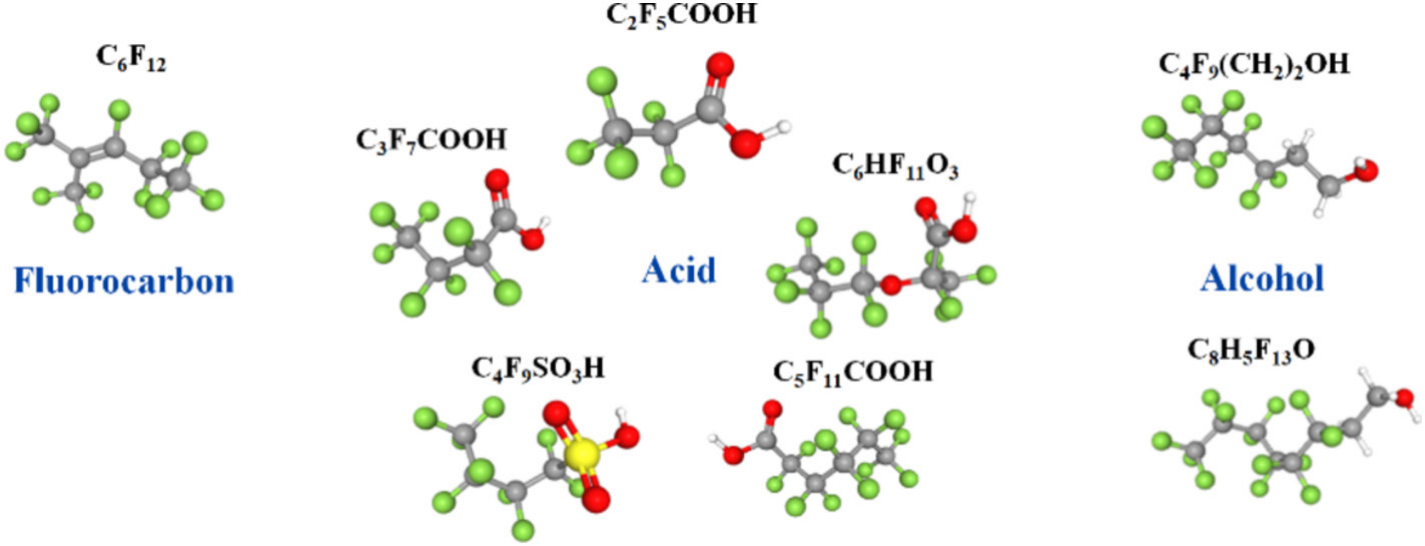
Representative chemical structures of grouped perfluoroalkyl and polyfluoroalkyl substance (PFAS) families.

The objective of the present study is to understand the kinetics of ion‐molecule reactions of H_3_O^+^, NO^+^, O_2_^+^•, OH^−^, O_2_^−^•, O^−^•, NO_2_^−^ and NO_3_^−^ with selected representative PFAS molecules: one fluorocarbon (perfluoro‐2‐methyl‐2‐pentene); five acids (pentafluoropropionic, heptafluorobutyric, nonafluorobutane‐1‐sulfonic, perfluorohexanoic and perfluoro(2‐methyl‐3‐oxahexanoic) acids); and two alcohols (nonafluoro‐1‐hexanol and tridecafluoro‐1‐octanol). The structures of these molecules are illustrated in Figure 1. Considering the complexity of the ion chemistry, we did not aim to develop an analytical method that could be characterised by a limit of detection.

## 2 Experimental

### 2.1 SIFT‐MS Instrument

**FIGURE 2:**
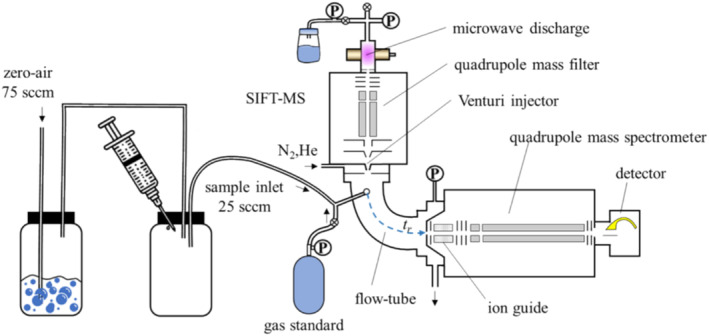
A schematic diagram of Voice200infinity. Reproduced from [2] under CC‐BY 4.0 open access licence.

A Voice200infinity SIFT‐MS instrument (Syft Technologies™, Christchurch, New Zealand) was used for this study (see Figure 2). This instrument (detailed elsewhere) [4] operates by generating the reagent ions H_3_O^+^, NO^+^, O_2_^+^, OH^−^, O_2_^−^•, O^−^•, NO_2_^−^ and NO_3_^−^, in a microwave discharge through a mixture of water and air. These ions are then filtered and introduced into the nitrogen carrier gas stream in the flow tube at a pressure of 400 mTorr and a temperature of 393 K. The experiments were conducted in an arrangement indicated in Figure 2. A sample containing PFAS vapour was injected using a syringe into a sample glass bottle flushed with clean zero‐air (continuously flowing at a rate of 75 sccm to gradually decrease the PFAS vapour concentration over time). To investigate the impact of humidity, we conducted additional experiments using a second bottle. In this setup, zero‐air flow was bubbled through water, resulting in a humidity level of approximately 2% water vapour content by volume (which is equivalent to the saturated vapour pressure at room temperature) before the air was introduced into the PFAS sample bottle (see Figure 2).

### 2.2 Reference Reagents

The following reagents were used for the present study as purchased from Sigma Aldrich (Darmstadt, Germany) with relative molecular masses (RMM) of the main isotopologue and the stated purities in parentheses: perfluoro‐2‐methyl‐2‐pentene (300 Da, > 98%), pentafluoropropionic acid (164 Da, 97%), heptafluorobutyric acid (214 Da, > 99.5%), nonafluorobutane‐1‐sulfonic acid (300 Da, 97%), perfluorohexanoic acid (314 Da, 97%), perfluoro(2‐methyl‐3‐oxahexanoic) acid (330 Da, 97%), nonafluoro‐1‐hexanol (264 Da, 97%) and tridecafluoro‐1‐octanol (364 Da, 97%).

### 2.3 Experiments to Determine Branching Ratios and Relative Rate Coefficients

Clean zero‐air was introduced at a flow rate of 75 sccm into a glass bottle, from which 25 sccm was sampled into the SIFT‐MS instrument, as indicated in Figure 2. Full scan mass spectra were repeatedly obtained in the mass‐to‐charge (*m/z*) range of 10 to 400 in the sequence of reagent ions generated in the different modes of operation of the ion source: [2] positive H_3_O^+^, NO^+^, O_2_^+^; negative wet OH^−^, O_2_^−^•; negative dry O^−^•, NO_2_^−^ and NO_3_^−^. The dwell time was 600 ms for each *m/z*, and the full cycle took 4 min. The appropriate volume of PFAS headspace was injected into the glass bottle (see Figure 2), which would decrease the reagent ion count rate noticeably, for which the PFAS concentration was then allowed to decrease over 90 min. The observed product ion intensity typically decreased from the initial > 10^5^ *c/s* to < 10^4^ *c/s*, this corresponds to approximate concentration of PFAS vapour from > 100 ppmv down to < 10 ppmv. The mass spectra were visually inspected and exported as images using the LabSyft software (version 1.8.1).

Product ion branching ratios and relative rate coefficients of reactions of different reagent ions with the same neutral compound were determined from the raw data using an algorithm implemented in Python. A similar approach has already been used [21]. Firstly, the experimental data in XML format were parsed and compiled into a peak table listing ion count rates for all reagent ions registered at different times. The process then involves selecting the 20 most intense signals from the raw data (based on the average count rate over the entire experiment), including the injected reagent ions, background signals and the dominant ions produced in the reactions. In the next step, the product ions were identified based on the decrease in signal intensity after introducing the reactant vapour. The background was then determined by extrapolating the ion count rates to the later time when the reactant vapour almost disappeared. To estimate the concentration of the neutral reactant molecules (M), the ratio of background‐corrected product to reagent ion signal (*P/R*) was used to provide an indirect measure of the concentration of M as:

(1) $$\left[M\right]=\ln\;\left(1+P/R\right)$$

The algorithm plotted the percentage of each product ion signal as a function of [M] to understand how the product ion distribution varied with [M]. Subsequently, parabolic curves were used to model the dependence of product ion percentages on [M], distinguishing between primary (linear fit) and secondary (parabolic fit) ion products. The secondary products, with intercept values less than 1%, were excluded from subsequent analyses to determine the true primary product ion branching ratios. Subsequently, a linear fit was applied to the data and branching ratios were taken as the intercept, when extrapolating to the limit of *P/R*, and thus [M] approaching zero.

Relative rate coefficients were determined by analysing changes in ion signals with varying concentrations of M, considering the increase in total product ion signals. The theoretical collisional rate coefficients (*k*_c_) were calculated using the Su and Chesnavich method [22]. The relative rates were then multiplied by *k*_c_ for the fastest observed reaction, giving the resulting experimental rate coefficient, *k*.

### 2.4 DFT Calculations

DFT calculations were performed using the ORCA 5.0.4 software [23]. Molecular geometries of all neutral reactant molecules and of all reagent and selected product ions were first drawn using the AVOGADRO software, followed by further optimisation using ORCA with the B3LYP DFT method with the 6‐311++G(d,p) basis set and D4 correction [24]. This level of theory was used to calculate the normal mode vibrational frequencies, total enthalpies, entropies, Gibbs free energies of the neutral molecules, their polarisabilities and dipole moments. Thermodynamic values were also calculated for the reagent ions, selected observed product ions and their corresponding neutral products. From these, the changes in enthalpies (∆*H*) and Gibbs free energies (∆*G*) were calculated for the reactions discussed later [25]. The accuracy of the results is considered to be within so‐called chemical accuracy (±1 kcal mol^−1^ = 4 kJ mol^−1^).

## 3 Results and Discussion

**FIGURE 3:**
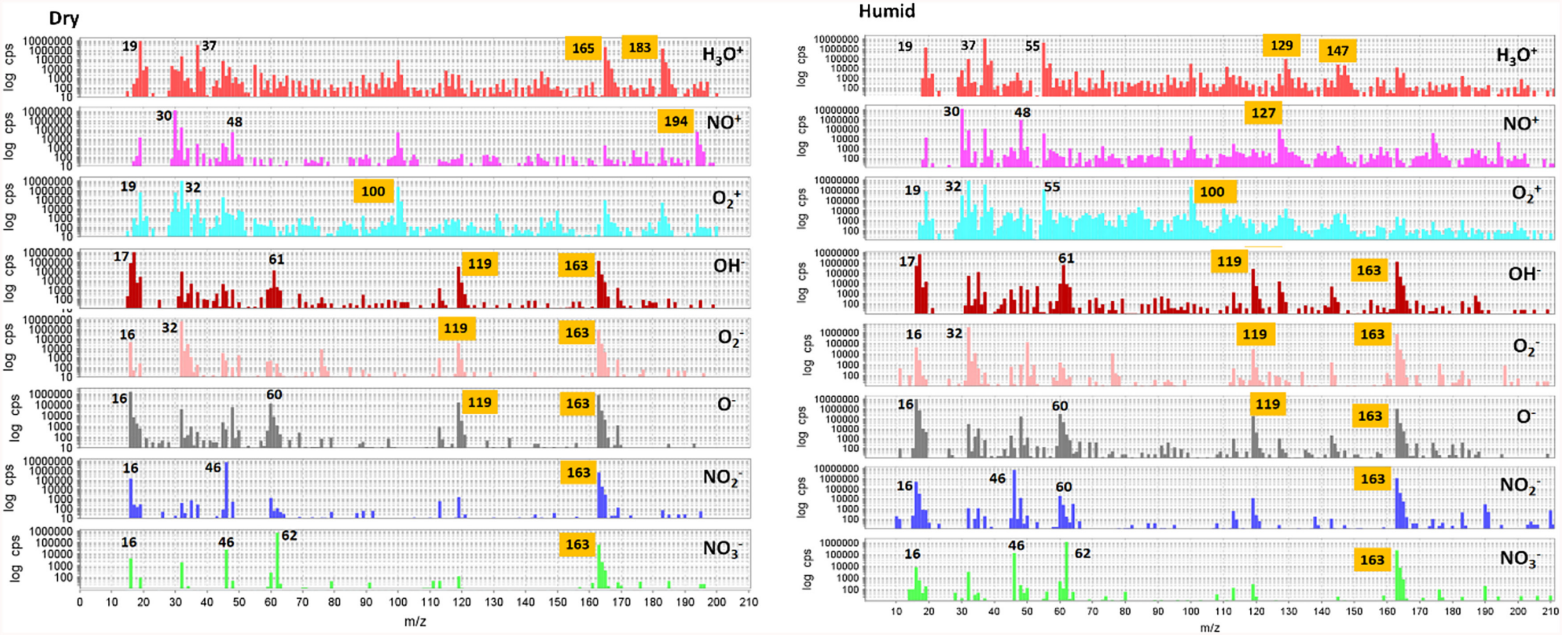
SIFT‐MS mass spectra obtained with all eight reagent ions for pentafluoropropionic acid (RMM 164 Da) in dry and humid conditions. The figure is compiled from images exported from the LabSyft software.

Figure 3 shows an example of some raw data obtained as full scan mass spectra for all eight reagent ions when pentafluoropropionic acid (RMM 164) vapour is introduced under dry (left) and humid conditions (right). We obtained more than a hundred such sets of spectra during the study, and the raw data are available in the repository. From these data, the most significant product ions were extracted based on their decreasing signal with a lowering concentration of PFAS vapour. Inspection of these raw mass spectra shows that the positive product ions are especially sensitive to humidity and that the negative reagent ions (including the usually less reactive NO_3_^−^) consistently react by proton extraction from the acid molecule.

### 3.1 Rate Coefficients

Table 1 gives the reaction rate coefficients obtained in the dry conditions from the increase of the relative total product ion signal (*P*/*R*) together with the molecular properties used for the calculation of *k*_c_; for each compound, the fastest reaction is assumed to proceed at *k* = *k*_c_. The data obtained in humid conditions indicate that the relative product ion signal from H_3_O^+^ decreases significantly because of the almost entire conversion of the product ions to H_3_O^+^H_2_O at *m/z* 37. The NO^+^ reactions are slow (except for those with the two alcohols), and they are not very sensitive to humidity. For the negative ions, associative detachment is likely responsible for the observation of a faster decay in O_2_^−^• reagent ions when compared with the increase of the product ion signals, as the free electrons are not detected. Also note that NO_2_^−^ and NO_3_^−^ only react with the acids and not with the fluorocarbons or the alcohols.

**TABLE 1:** The experimentally derived reaction rate coefficients (*k*) and the theoretical collisional rate coefficients (*k*_c_, shown in square brackets) of the PFAS reactions obtained in N_2_ carrier gas at 393 K under dry conditions. The units of *k* and *k*_c_ are 10^−9^ cm^3^ s^−1^.

Compound (RMM)	Polarisability *α,* (Å, 10^−24^ cm)	Dipole moment D, Debye	*k*, [*k*_c_] (× 10^−9^ cm^3^ s^−1^)							
			H_3_O^+^	NO^+^	O_2_^+^•	O^−^•	OH^−^	O_2_^−^•	NO_2_^−^	NO_3_^−^
Perfluoro‐2‐methyl‐2‐pentene (300)	11.7	0.7	0.1 [2.1]	0.0 [1.7]	1.4 [1.6]	2.3 [2.3]	0.4 [2.2]	0.4 [1.6]	0.4 [1.4]	0.0 [1.2]
Pentafluoropropionic acid (164)	6.8	2.4	1.9 [2.9]	0.0 [2.4]	1.2 [2.4]	3.1 [3.1]	0.7 [3.1]	0.8 [2.4]	0.6 [2.0]	0.7 [1.8]
Heptafluorobutyric acid (214)	8.6	2.3	2.2 [2.9]	0.1 [2.4]	0.9 [2.4]	3.2 [3.2]	0.7 [3.1]	0.7 [2.4]	0.6 [2.0]	0.8 [1.8]
Nonafluorobutane‐1‐sulfonic acid (300)	12.3	2.8	2.6 [3.4]	0.2 [2.8]	1.4 [2.7]	3.7 [3.7]	1.0 [3.6]	1.0 [2.7]	0.9 [2.3]	2.0 [2.0]
Perfluorohexanoic acid (314)	12.3	2.2	2.3 [3.0]	0.1 [2.4]	2.3 [2.3]	2.5 [3.2]	2.1 [3.1]	2.1 [2.3]	1.8 [2.0]	1.7 [1.7]
Perfluoro(2‐methyl‐3‐oxahexanoic) acid (330)	13.0	2.5	2.2 [3.2]	0.2 [2.6]	1.8 [2.5]	3.5 [3.5]	0.8 [3.4]	0.7 [2.5]	0.6 [2.2]	0.6 [1.9]
Nonafluoro‐1‐hexanol (264)	12.0	2.0	2.8 [2.8]	1.0 [2.3]	1.3 [2.2]	1.5 [3.1]	0.7 [3.0]	0.6 [2.2]	0.0 [1.9]	0.0 [1.7]
Tridecafluoro‐1‐octanol (364)	15.8	2.1	2.2 [3.1]	1.0 [2.5]	2.4 [2.4]	1.9 [3.3]	1.2 [3.2]	0.5 [2.4]	0.0 [2.0]	0.0 [1.8]

### 3.2 Branching Ratios

Table 2 gives the product ions identified for the reactions of the three positive reagent ions with the tested PFAS substances, and Table 3 shows the product ions from the reaction with the negative reagent ions. For each product ion, the assigned formula is given together with the observed *m/z* for which the branching ratio is obtained under dry conditions. The symbols ‘<’ and ‘>’ indicate significant changes with humidity (from dry zero air to 2% water vapour by volume). Generally, the product ions of the H_3_O^+^ and NO^+^ reactions with PFAS disappear under humid conditions, as indicated by the ‘<’ symbols, as they are entirely converted to the reagent ion hydrates H_3_O^+^H_2_O and NO^+^H_2_O, as indicated by the ‘>’ symbols.

**TABLE 2:** Product ions for the reactions of the positive reagent ions with the PFAS observed using the Voice200infinity with a N_2_ carrier gas at a flow tube temperature of 393 K. The relative molecular mass (RMM) of the PFAS is shown in parentheses after the PFAS name; for the product ions, the assigned formula along with the *m/z* value of the product ion is shown first, followed by the branching ratio.

Compound RMM, formula	H_3_O^+^			NO^+^			O_2_^+^•		
	Product	*m/z*	b_r_	Product	*m/z*	b_r_	Product	*m/z*	b_r_
Perfluoro‐2‐methyl‐2‐pentene (300) C_6_F_12_	(M‐F) ^+^	281	< 66%	—	—	—	C_4_F_7_^+^	181	46%
	C_6_F_9_O^+^	259	> 16%				C_5_F_9_^+^	231	25%
	C_6_HF_10_O^+^	279	< 8%				C_4_F_5_O^+^	159	19%
Pentafluoropropionic acid (164) C_3_HF_5_O_2_	MH^+^	165	< 40%	MNO^+^	194	< 40%	C_2_F_4_^+^•	100	80%
	MH_3_O^+^	183	< 25%	H_2_ONO^+^	48	> 60%	H_3_O+	19	20%
	H_3_O^+^H_2_O	37,55	> 35						
Heptafluorobutyric acid (214) C_4_HF_7_O_2_	MH^+^	215	< 20%	MNO^+^	244	< 45%	C_3_F_6_^+^•	150	80%
	MH_3_O^+^	233	< 16%	H_2_ONO^+^	48	> 55%	H_3_O+	19	20%
	H_3_O^+^H_2_O	37,55	> 64%						
Nonafluorobutane‐1‐sulfonic acid (300) C_4_H F_9_SO_3_	MH^+^	301	< 5%	MNO^+^	330	< 70%	H_3_O^+^	19	80%
	MH_3_O^+^	319	< 25%	H_2_ONO^+^	48	> 30%	SO_2_H^+^	65	20%
	H_3_O^+^H_2_O	37	> 70%						
Perfluorohexanoic acid (314) C_6_HF_11_O_2_	MH^+^	315	< 30%	MNO^+^	344	< 14%	C_5_F_10_^+^•	250	50%
	MH_3_O^+^	333	< 70%	H_2_ONO^+^	48	> 86%	H_3_O^+^	19	20%
	H_3_O^+^H_2_O	37,55	> 0%				C_3_F_5_^+^	131	30%
Perfluoro(2‐methyl‐3‐oxahexanoic) acid (330) C_6_HF_11_O_3_	MH^+^	331	< 22%	MNO^+^	360	< 85%	C_5_F_10_O^+^•	266	85%
	MH_3_O^+^	349	< 33%	H_2_ONO^+^	48	> 15%	H_3_O^+^	19	15%
	H_3_O^+^H_2_O	37	> 45%						
Nonafluoro‐1‐hexanol (264) C_6_F_9_H_4_OH	MH^+^	265	< 25%	MNO^+^	294	< 100%	CH_3_O^+^	31	< 60%
	MH_3_O^+^	283	> 75%	H_2_ONO^+^	48	> 0%	C_5_H_3_F_7_^+^•	196	< 40%
							H_3_O^+^H_2_O	37,55	> 0%
Tridecafluoro‐1‐octanol (364) C_8_H_4_F_13_OH	MH^+^	365	25%	MNO^+^	394	< 100%	C_7_H_3_F_11_^+^	296	60%
	MH_3_O^+^	383	75%	H_2_ONO^+^	48	> 0%	CH_3_O^+^	31	40%

*Note:* “—” indicates no reaction.

**TABLE 3:** Product ions for the reactions of the negative reagent ions with the PFAS observed using the Voice200infinity with a N_2_ carrier gas at a flow tube temperature of 393 K. The RMM of the PFAS is shown in parentheses after the PFAS name; for the product ions, the assigned formula along with the *m/z* value of the product ion is shown first, followed the branching ratio.

Compound RMM, formula	O^−^˙			OH^−^			O_2_^−^•			NO_2_^−^			NO_3_^−^		
	Product	*m/z*	b_r_	Product	*m/z*	b_r_	Product	*m/z*	b_r_	Product	*m/z*	b_r_	Product	*m/z*	b_r_
Perfluoro‐2‐methyl‐2‐pentene (300) C_6_F_12_	C_4_F_7_O^−^	197	65%	C_6_F_11_O^−^	297	65%	C_6_F_12_^−^•	300	62%	C_3_F_6_NO^−^	180	100%	—	—	—
	C_5_F_9_O^−^	247	29%	C_3_F_5_^−^	131	19%	?	198	27%	C_6_F_4_O_2_^−^					
	C_3_F_5_^−^	131	7%	C_4_F_7_^−^	181	16%	C_3_F_6_O^−^•	166	11%						
Pentafluoropropionicacid (164) C_3_HF_5_O_2_	(M‐H)^−^	163	85%	(M‐H)^−^	163	80%	(M‐H)^−^	163	96%	(M‐H)^−^	163	100%	(M‐H)^−^	163	100%
	C_2_F_5_^−^	119	15%	C_2_F_5_^−^	119	20%	C_2_F_5_^−^	119	4%						
Heptafluorobutyric acid (214) C_4_HF_7_O_2_	(M‐H)^−^	213	77%	(M‐H)^−^	213	74%	(M‐H)^−^	213	88%	(M‐H)^−^	213	100%	(M‐H)^−^	213	82%
	C_3_F_7_^−^	169	23%	C_3_F_7_^−^	169	26%	C_3_F_7_^−^	169	12%				C_4_HF_7_NO_5_^−^	276	18%
Nonafluorobutane‐1‐sulfonic acid (300) C_4_H F_9_SO_3_	(M‐H)^−^	299	100%	(M‐H)^−^	299	100%	(M‐H)^−^	299	100%	(M‐H)^−^	299	100%	(M‐H)^−^	299	100%
Perfluorohexanoic acid (314) C_6_HF_11_O_2_	(M‐H)^−^	313	75%	(M‐H)^−^	313	75%	(M‐H)^−^	313	82%	(M‐H)^−^	313	92%	(M‐H)^−^	313	74%
	C_5_F_11_^−^	269	25%	C_5_F_11_^−^	269	25%	C_5_F_11_^−^	269	18%	C_5_F_11_^−^	269	8%	C_5_F_11_^−^	269	26%
Perfluoro(2‐methyl‐3‐oxahexanoic) acid (330) C_6_HF_11_O_3_	C_5_F_11_O^−^	285	79%	C_5_F_11_O^−^	285	81%	C_5_F_11_O^−^	285	82%	C_5_F_11_O^−^	285	81%	C_5_F_11_O^−^	285	76%
	C_3_F_7_^−^	169	18%	C_3_F_7_^−^	169	15%	C_3_F_7_^−^	169	15%	C_3_F_7_^−^	169	13%	C_3_F_7_^−^	169	10%
	(M‐H)^−^	329	3%	(M‐H)^−^	329	3%	(M‐H)^−^	329	3%	(M‐H)^−^	329	6%	(M‐H)^−^	329	14%
Nonafluoro‐1‐hexanol (264) C_6_F_9_H_4_OH	C_6_H_2_F_9_O^−^	261	< 65%	HF_2_^−^	39	< 60%	C_6_H_5_F_9_O_3_^−^•	296	100%	—	—	—	—	—	—
	C_2_H_4_FO^−^	63	23%	C_2_H_4_FO^−^	63	22%									
	C_6_HF_8_O^−^	241	< 12%	C_6_H_2_F_7_O^−^	223	9%									
		19,37	> 0%	C_5_H_4_F_7_O^−^	213	< 8%									
					19,37	> 0%									
Tridecafluoro‐1‐octanol (364) C_8_H_4_F_13_OH	HF_2_^−^	39	< 47%	HF_2_^−^	39	< 65%	C_8_H_5_F_13_O_3_^−^•	396	100%	—	—	—	—	—	—
	C_8_H_2_F_13_O^−^	361	< 37%	C_7_H_4_F_11_O^−^	313	< 23%									
	C_7_H_4_F_11_O^−^	313	17%	C_7_H_3_F_10_O^−^	293	< 13%									

*Note:* “—” indicates no reaction.

#### 3.2.1 Positive ion Chemistry

The H_3_O^+^ reaction with a fluorocarbon results in the formation of the (M‐F)^+^ ion as the main reaction channel.

(2) $$H_{3}O^{+}+C_{6}F_{12}\to {C_{6}F_{11}}^{+}+H_{3}\mathrm{FO}$$

Additional fragments were observed as minor channels (see Table 2). Although the experiment clearly confirms that reaction (2) proceeds at *k* = 0.05 *k*_c_, we cannot find a structure from DFT calculations that would render this reaction exothermic or exergonic. Calculated Δ*G* and Δ*H* are positive for all structures resulting from removing an F^−^ ion from different sites of the neutral molecule (see Table 4). However, as the experiment clearly confirms the formation of C_6_F_11_^+^, its structure must be rearranged to a different lower total energy configuration. The product ions remain detectable even under humid conditions, albeit at a somewhat different branching ratio.

**TABLE 4:** Results of the DFT calculations of reaction enthalpy (Δ*H*) and Gibbs free energy (Δ*G*) changes for the H_3_O^+^ reactions indicated.

PFAS compound	Product ion	*m/z*	Δ*H* (kJ/mol)	Δ*G* (kJ/mol)
Perfluoro‐2‐methyl‐2‐pentene	[M‐F] ^+^	300	52	32
Pentafluoropropionic acid	MH^+^	164	−10	−11
Heptafluorobutyric acid	MH^+^	214	−17	−13
Nonafluorobutane‐1‐sulfonic acid	MH^+^	300	−8	−10
Perfluorohexanoic acid	MH^+^	314	−25	−21
Perfluoro(2‐methyl‐3‐oxahexanoic) acid	MH^+^	330	4	7
Nonafluoro‐1‐hexanol	MH^+^	264	−42	−40
Tridecafluoro‐1‐octanol	MH^+^	364	−42	−40

For the remaining PFAS, acids and alcohols, the main products observed for the reactions with H_3_O^+^ are the protonated molecules, MH^+^, and the adduct ions, MH_3_O^+^:

(3a) $$H_{3}O^{+}+M\to MH^{+}+H_{2}O$$

(3b) $$\overset{\;N_{2\;}}{\to }\;MH_{3}O^{+}$$

These product ions undergo fast secondary reactions with H_2_O molecules, resulting in the formation of H_3_O^+^H_2_O at *m/z* 37:

(4) $${\mathrm{MH}}_{3}O^{+}+H_{2}O\to H_{3}O^{+}H_{2}O+M$$

Results of the DFT calculations for the lowest energy structures of the (MH)^+^ product ions of the Δ*H* and Δ*G* are given in Table 4. Note here that the calculations indicate that almost all the observed reactions (3a) are exothermic proton transfer and both Δ*H* and Δ*G* are negative. A notable exception is for perfluoro(2‐methyl‐3‐oxahexanoic) acid, for which the calculated Δ*H* and Δ*G* are positive but may be considered to be near 0 within the combined calculation accuracy. It is possible that the etheric O atom may cause more complex rearrangements of the protonated molecule than we considered in our DFT structures.

For NO^+^, the fluorocarbon shows no reaction, presumably because there is no stable bond formed between NO^+^ and M, this agrees with previous studies where NO^+^ did not react with similar C_2_F_4_, C_3_F_6_ and 2‐C_4_F_8_ fluorocarbons [26]. However, the acids and alcohols react by association:

(5) $$NO^{+}+M\;\overset{\;\;N_{2\;}}{\to }\;\mathrm{MN}O^{+}$$

A ligand‐switching reaction with H_2_O molecules follows this. Thus, under humid conditions, the adduct product ion, MNO^+^, decreases significantly because of the conversion to H_2_ONO^+^ at *m/z* 48, (see Table 2).

(6) $$\mathrm{MN}O^{+}+H_{2}O\to H_{2}\mathrm{ON}O^{+}+M$$

O_2_^+^• reactions with all eight PFAS compounds result in the formation of various fragment ions.

(7) $${O_{2}}^{+}˙+M\to {\left(M-R\right)}^{+}+R˙+O_{2}$$

The percentages of the products listed in Table 2 do not show any obvious pattern. It is interesting to note that in the case of acids, H_3_O^+^ appears to be produced as an ionic product. However, because there is only one hydrogen atom in the acid molecules, this indicates that these must involve secondary proton transfer reactions of hydrogen‐containing reactive ion products, which can be symbolically expressed as follows:

(8) $$AH^{+}+H_{2}O\to H_{3}O^{+}+A$$

#### 3.2.2 Negative ion Chemistry

The experiments carried out in dry conditions provide data on the primary reactions of the reagent ions with PFAS molecules. All the data are given in Table 3, for which some observations are interesting to discuss.

The reaction of O^−^• with the C_6_F_12_ fluorocarbon molecules proceeds via the incorporation of the O atom into the molecule, resulting in the formation of the C_4_F_7_O^−^ ion at *m/z* 197, as the main product:

(9) $$O^{-}˙+C_{6}F_{12}\to C_{4}F_{7}O^{-}+C_{2}F_{5}˙$$

with other minor fragment ions also formed.

Similarly, the main channel in the reaction of OH^−^ with the fluorocarbon results in the formation of the C_6_F_11_O^−^ ion at *m/z* 297:

(10) $${\mathrm{OH}}^{-}+C_{6}F_{12}\to C_{6}F_{11}O^{-}+\mathrm{HF}$$

where the neutral product is the stable hydrogen fluoride molecule.

O_2_^−^• reacts with the fluorocarbon by electron transfer, forming the molecular radical anion C_6_F_12_^−^• at *m/z* 300, accompanied by minor fragments.

(11) $${O_{2}}^{-}˙+C_{6}F_{12}\to C_{6}{F_{12}}^{-}˙+O_{2}$$

The minor product ion observed at *m/z* 198 remains unexplained; it cannot be a primary product but could result from a fast reaction of one of the primary products with N_2_, residual CO_2_ or H_2_O molecules.

The O^−^•, OH^**−**^ and O_2_^−^• ions react with acids by accepting a proton transferred from the PFAS molecules, forming (M‐H)^−^ and other minor product ions (see Table 3). For example, the major product formed in reactions involving O^−^•, OH^**−**^ and O_2_^−^• with pentafluoropropionic acid is (M‐H)^−^ at *m/z* 163:

(12) $$\begin{array}{c} O^{-}˙/{\mathrm{OH}}^{-}/{O_{2}}^{-}˙+C_{2}F_{5}\text{COOH}\to C_{2}F_{5}{\mathrm{COO}}^{-\;}\\ +\;\mathrm{OH}˙/H_{2}O/{\mathrm{HO}}_{2}˙ \end{array}$$

O^−^• and OH^**−**^ reagent ion reactions with PFAS alcohol molecules are relatively slow, being well below the collisional rates and result in multiple primary product ions forming. Observation of HF_2_^−^ bifluoride as a product ion at *m/z* 39 is interesting. For example:

(13) $${\mathrm{OH}}^{-}+C_{8}F_{13}H_{4}\mathrm{OH}\to {{\mathrm{HF}}_{2}}^{-}+C_{8}H_{5}F_{11}O_{2}$$

We cannot be certain about the neutral product, but it could be perfluorooctanoic acid.

O_2_^−^• reacts with these PFAS alcohols by association, forming adduct ions observed at *m/z* 296 for nonafluoro‐1‐hexanol and at *m/z* 396 for tridecafluoro‐1‐octanol:

(14) $${O_{2}}^{-}˙+C_{6}F_{9}H_{4}\mathrm{OH}\to C_{6}H_{5}F_{9}{O_{3}}^{-}˙$$

(15) $${O_{2}}^{-}˙+C_{8}F_{13}H_{4}\mathrm{OH}\to C_{8}H_{5}F_{13}{O_{3}}^{-}˙$$

Finally, note that NO_2_^−^ and NO_3_^−^ ions do not observably react with alcohols. However, they react with acids by proton transfer, similarly to reaction (12), as indicated in Table 3.

Under humid conditions, secondary reactions with water molecules can take place. Interestingly, unlike positive ion chemistry, the products of these anion reactions remain largely unchanged. The rate coefficients are also unaffected, indicating that humidity would not influence this ionisation in SIFT‐MS significantly. This trend holds true for both acidic and fluorocarbon compounds, where the observed products are consistent regardless of moisture. However, notable changes are observed for O^−^• and OH^−^ ions for alcohols, indicating the reactivity of product ions with H_2_O molecules. Interestingly, the adducts formed from the reaction of O_2_^−^• with alcohols do not exhibit significant changes in response to humidity.

## 4 Conclusion

Although PFAS are often considered a consistent group of compounds because of their environmental importance and toxicity, their behaviour in gas‐phase ion chemistry is highly variable and (as observed in this study) dominated by their chemical nature, whether they are fluorocarbons, acids, or alcohols. This variability is critical for developing SIFT‐MS analytical methods, as each subgroup exhibits unique ion chemistry [9]. Our findings indicate that the negative reagent ions, particularly O^−^•, OH^−^ and O_2_^−^•, have predictable reactivities unaffected by sample humidity, making them more reliable for the practical SIFT‐MS analyses of PFAS in humid matrices. In contrast, positive product ions are overly sensitive to humidity, complicating (and almost preventing) their use in SIFT‐MS analyses under humid conditions.

The methodology based on the automated interpretation of full‐scan SIFT‐MS data for eight reagent ions in a rapid cycle represents an important advance. This approach is ready to be applied to further studies across various classes of SIFT‐MS analytes.

Integrating these ion chemistry findings and data into a routine SIFT‐MS library could facilitate PFAS detection and quantification. Future work is needed to expand on this methodology by including a wider range of PFAS subtypes and exploring the potential of machine learning in the automation and refinement of SIFT‐MS data processing for complex mixtures. Also, the analytical methods, including the appropriate choice of the reagent anions and the most robust product ions, must be developed for specific matrices before the LOD can be quoted.

## Author Contributions

**Stefan James Swift:** conceptualisation, investigation, formal analysis, writing – original draft. **Maroua Omezzine Gnioua:** investigation, validation, formal analysis, visualisation, writing – review and editing. **Kseniya Dryahina:** conceptualisation, project administration, writing – review and editing. **Patrik Španěl:** conceptualisation, funding acquisition, methodology, software, writing – original draft, writing – review and editing.

## Conflicts of Interest

The authors declare no conflicts of interest.

## Data Availability

The data supporting this article are available in the National Repository at https://doi.org/10.48700/datst.fkw19‐mxb81 under the Creative Commons open‐access licence.

## References

[1] D. Smith , P. Španěl , N. Demarais , V. S. Langford , and M. J. McEwan , “Recent Developments and Applications of Selected ion Flow Tube Mass Spectrometry, SIFT‐MS,” Mass Spectrometry Reviews (2023): e21835. 36776107 10.1002/mas.21835 PMC11792439

[2] V. S. Langford , K. Dryahina , and P. Španěl , “Robust Automated SIFT‐MS Quantitation of Volatile Compounds in Air Using a Multicomponent Gas Standard,” Journal of the American Society for Mass Spectrometry 34, no. 12 (2023): 2630–2645. 37988479 10.1021/jasms.3c00312 PMC10704587

[3] D. Smith , M. J. McEwan , and P. Španěl , “Understanding Gas Phase Ion Chemistry Is the Key to Reliable Selected Ion Flow Tube‐Mass Spectrometry Analyses,” Analytical Chemistry 92, no. 19 (2020): 12750–12762. 32857492 10.1021/acs.analchem.0c03050

[4] S. J. Swift , P. Španěl , N. Sixtová , and N. Demarais , “How to Use Ion‐Molecule Reaction Data Previously Obtained in Helium at 300 K in the new Generation of Selected ion Flow Tube Mass Spectrometry Instruments Operating in Nitrogen at 393 K,” Analytical Chemistry 95, no. 29 (2023): 11157–11163. 37454354 10.1021/acs.analchem.3c02173 PMC10372871

[5] P. Španěl , S. J. Swift , K. Dryahina , and D. Smith , “Relative Influence of Helium and Nitrogen Carrier Gases on Analyte ion Branching Ratios in SIFT‐MS,” International Journal of Mass Spectrometry 476 (2022): 116835.

[6] D. Hera , V. S. Langford , M. J. McEwan , T. I. McKellar , and D. B. Milligan , “Negative Reagent Ions for Real Time Detection Using SIFT‐MS,” Environments 4, no. 1 (2017): 16.

[7] J. Glüge , M. Scheringer , I. T. Cousins , et al., “An Overview of the Uses of Per‐ and Polyfluoroalkyl Substances (PFAS),” Environmental Science: Processes & Impacts 22, no. 12 (2020): 2345–2373. 33125022 10.1039/d0em00291g PMC7784712

[8] Z. Abunada , M. Y. D. Alazaiza , and M. J. K. Bashir , “An Overview of Per‐ and Polyfluoroalkyl Substances (PFAS) in the Environment: Source, Fate, Risk and Regulations,” Watermark 12, no. 12 (2020): 3590.

[9] C. F. Kwiatkowski , D. Q. Andrews , L. S. Birnbaum , et al., “Scientific Basis for Managing PFAS as a Chemical Class,” Environmental Science & Technology Letters 7, no. 8 (2020): 532–543. 34307722 10.1021/acs.estlett.0c00255 PMC8297807

[10] B. Rudzanová , V. Thon , H. Vespalcová , et al., “Altered Transcriptome Response in PBMCs of Czech Adults Linked to Multiple PFAS Exposure: B Cell Development as a Target of PFAS Immunotoxicity,” Environmental Science & Technology 58, no. 1 (2024): 90–98. 38112183 10.1021/acs.est.3c05109 PMC10785749

[11] E. Panieri , K. Baralic , D. Djukic‐Cosic , A. Buha Djordjevic , and L. Saso , “PFAS Molecules: A Major Concern for the Human Health and the Environment,” Toxics 10 (2022): 44, 10.3390/toxics10020044. 35202231 PMC8878656

[12] Y. N. Liu , A. D. Pereira , and J. W. Martin , “Discovery of C_5_‐C_17_ Poly‐ and Perfluoroalkyl Substances in Water by In‐Line SPE‐HPLC‐Orbitrap With in‐Source Fragmentation Flagging,” Analytical Chemistry 87, no. 8 (2015): 4260–4268. 25818392 10.1021/acs.analchem.5b00039

[13] T. P. Riedel , J. R. Lang , M. J. Strynar , A. B. Lindstrom , and J. H. Offenberg , “Gas‐Phase Detection of Fluorotelomer Alcohols and Other Oxygenated Per‐ and Polyfluoroalkyl Substances by Chemical Ionization Mass Spectrometry,” Environmental Science & Technology Letters 6, no. 5 (2019): 289–293. 31179348 10.1021/acs.estlett.9b00196 PMC6550326

[14] M. J. Davern , G. V. West , C. M. A. Eichler , B. J. Turpin , Y. Zhang , and J. D. Surratt , “External Liquid Calibration Method for Iodide Chemical Ionization Mass Spectrometry Enables Quantification of Gas‐Phase Per‐ and Polyfluoroalkyl Substances (PFAS) Dynamics in Indoor Air,” Analyst 149, no. 12 (2024): 3405–3415. 38712891 10.1039/d4an00100a

[15] B. B. Bowers , J. A. Thornton , and R. C. Sullivan , “Evaluation of Iodide Chemical Ionization Mass Spectrometry for Gas and Aerosol‐Phase Per‐ and Polyfluoroalkyl Substances (PFAS) Analysis,” Environmental Science: Processes & Impacts 25, no. 2 (2023): 277–287. 36189623 10.1039/d2em00275b

[16] C. J. Young , S. Joudan , Y. Tao , J. J. B. Wentzell , and J. Liggio , “High Time Resolution Ambient Observations of Gas‐Phase Perfluoroalkyl Carboxylic Acids: Implications for Atmospheric Sources,” Environmental Science & Technology Letters 11, no. 12 (2024): 7.

[17] R. A. Morris , “Gas‐Phase Reactions of Oxide and Superoxide Anions With CF_4_, CF_3_Cl, CF_3_Br, CF_3_I, and C_2_F_4_ at 298 K and 500 K,” Journal of Chemical Physics 97, no. 4 (1992): 2372–2381.

[18] A. J. Bell , C. J. Hayhurst , C. A. Mayhew , and P. Watts , “On the Reactions of Perfluoroisobutene With Some Anions in the Gas‐Phase—Studies in an Ion Mobility Spectrometer,” International Journal of Mass Spectrometry and Ion Processes 140 (1994): 133–147.

[19] E. P. F. Lee , J. M. Dyke , and C. A. Mayhew , “Study of the OH^−^+CH_2_F_2_ Reaction by Selected Ion Flow Tube Experiments and Ab Initio Calculations,” Journal of Physical Chemistry. A 102, no. 43 (1998): 8349–8354.

[20] P. Watts , R. A. Kennedy , P. Španěl , M. Omezzine Gnioua , and C. A. Mayhew , “The Gas Phase Reactions of Some Fluorinated Ethenes With Several Anions,” International Journal of Mass Spectrometry (forthcoming).

[21] M. Omezzine Gnioua , S. J. Swift , and P. Španěl , “Selected Ion Flow Tube Studies of the Reactions of H_3_O^+^, NO^+^, O_2_ ^+^˙ and O^−^˙ Ions With Alkanes in He and N_2_ Carrier Gases at Different Temperatures,” Physical Chemistry Chemical Physics 26, no. 41 (2024): 26585–26593. 39400284 10.1039/d4cp03105a

[22] T. Su and W. J. Chesnavich , “Parametrization of the Ion‐Polar Molecule Collision Rate‐Constant by Trajectory Calculations,” Journal of Chemical Physics 76, no. 10 (1982): 5183–5185.

[23] F. Neese , “Software Update: The ORCA Program System—Version 5.0,” WIREs Computational Molecular Science 12, no. 5 (2022): e1606.

[24] E. Caldeweyher , S. Ehlert , A. Hansen , et al., “A Generally Applicable Atomic‐Charge Dependent London Dispersion Correction,” Journal of Chemical Physics 150, no. 15 (2019): 154122. 31005066 10.1063/1.5090222

[25] S. J. Swift , N. Sixtova , M. Omezzine Gnioua , and P. Španěl , “A SIFT‐MS Study of Positive and Negative ion Chemistry of the Ortho‐, Meta‐ and Para‐Isomers of Cymene, Cresol, and Ethylphenol,” Physical Chemistry Chemical Physics 25, no. 27 (2023): 17815–17827. 37377058 10.1039/d3cp02123h

[26] G. K. Jarvis , C. A. Mayhew , and R. P. Tuckett , “Study of the Gas Phase Reactions of Several Perfluorocarbons With Positive Ions of Atmospheric Interest,” Journal of Physical Chemistry 100, no. 43 (1996): 17166–17174.

